# Determinants of placental iodine concentrations in a mild-to-moderate iodine-deficient population: an ENVIR*ON*AGE cohort study

**DOI:** 10.1186/s12967-020-02601-8

**Published:** 2020-11-10

**Authors:** Kristof Y. Neven, Bianca Cox, Karen Vrijens, Michelle Plusquin, Harry A. Roels, Ann Ruttens, Tim S. Nawrot

**Affiliations:** 1 Centre for Environmental Sciences, Hasselt University, Agoralaan Gebouw D, 3590 Diepenbeek, Belgium; 2 Louvain Centre for Toxicology and Applied Pharmacology, Université Catholique de Louvain, E. Mounierlaan 53, 1200 Brussels, Belgium; 3 Sciensano, SD Chemical, and Physical Health Risks, Leuvensesteenweg 17, 3080 Tervuren, Belgium; 4 Department of Public Health & Primary Care, Leuven University, Kapucijnenvoer 35, 3000 Leuven, Belgium

**Keywords:** Neonates, Placenta, Iodine, Weight gain, Alcohol, BMI, Seasonality, Thyroid hormones

## Abstract

**Background:**

Iodine is an essential trace element for the production of thyroid hormones, and plays a key role during the gestational period for optimal foetal growth and (neuro-)development. To this day, iodine deficiency remains a global burden. Previous studies indicate that the placenta can store iodine in a concentration-dependent manner and serve as a long-term storage supply, but studies on the determinants of long-term placental iodine load are limited.

**Methods:**

The placental iodine concentrations were determined for 462 mother-neonate pairs from the ENVIRONAGE birth cohort (Limburg, Belgium). Sociodemographic and clinical variables were obtained from questionnaires and medical files. Determinants of placental iodine concentration were identified using stepwise multiple regression procedures (*p* value < 0.15). The biological significance of our findings was investigated by measuring the plasma thyroid hormones in maternal and cord blood of 378 participants.

**Results:**

A higher pre-pregnancy BMI, higher gestational weight gain, and alcohol consumption during pregnancy were linked with lower placental iodine storage. Multi-vitamin supplementation during pregnancy and longer gestation were associated with higher levels of placental iodine. Children born during the winter period had on average higher placental iodine levels. Besides, we found a significant positive time trend for placental iodine load over the study period 2013 to 2017. Lastly, we observed positive associations of both the maternal and cord plasma thyroxine concentrations with placental iodine load, emphasizing their biological link.

**Conclusions:**

This study identified some determinants likely presenting a risk of reduced iodine storage during the gestational period of life. Future studies should elucidate the effects of lower placental iodine load on neonatal health, and health later in life.

## Background

Iodine is an essential trace element that plays a vital role in the production and regulation of thyroid-stimulating hormone (TSH) and the thyroid hormones tri-iodothyronine (FT_3_) and thyroxine (FT_4_), which are essential for optimal foetal growth and (neuro) development [1]. Previous findings of the Avon Longitudinal Study of Parents and Children (ALSPAC) indicate that mild deficiency in iodine during the gestational period, measured as maternal urinary iodine concentrations, was linked with a lower verbal IQ status at the age of 8 years and worse reading comprehension at the age of 9 years [2]. Furthermore, the prevalence of attention deficit and hyperactivity disorders is higher in the offspring of iodine-deficient populations [3]. While numerous iodine supplementation programmes have been implemented over the last decade, still, an estimated 2.2 billion individuals (~ 38% of the world’s population) are affected by iodine deficiency [4], which remains a leading cause of preventable mental retardation worldwide [5].

Recently, it has been shown that the placenta can store iodine in a concentration-dependent manner [6, 7], suggesting that this storage capacity could protect both foetus and mother from temporary inadequacies in maternal dietary iodine intake. Placental iodine is likely to better reflect long-term iodine intake, whereas the urinary iodine concentrations mainly reflect short-term intake [8].

Previous studies indicate that several variables, such as seasonal sampling and gestational duration, are linked with urinary iodine concentrations. However, no study to date has linked these variables to placental iodine levels. As placental iodine concentrations may be representative of the iodine intake over the entire gestational period, it would be less subject to dietary fluctuations compared to the urinary concentrations and may better reflect the in utero exposure to this essential element. The present cross-sectional study aims to evaluate the importance of the selected variables on the placental iodine levels.

## Methods

### Study design

The ENVIRonmental influences *ON* early AGEing (ENVIR*ON*AGE) birth cohort was established in 2011 at Hasselt University and recruits pairs of mothers and neonates (singleton births only) at birth at the East-Limburg Hospital (Genk, Belgium). The catchment area of the cohort is located in the north-east of Belgium, in the province of Limburg, Flanders [9]. A nation-wide survey of Belgian pregnant women showed that nearly 60% was iodine deficient [10], which corroborated with findings in the ENVIR*ON*AGE cohort [8]. The Ethics Committee of Hasselt University and the East-Limburg Hospital approved the study protocol that was carried out following the Declaration of Helsinki. Written informed consent was obtained from all participating mothers.

The inclusion criteria were mothers who provided informed consent and were able to fill out questionnaires in Dutch, while planned caesareans were excluded. Participants were recruited equally during all seasons of the year, and midwives recorded the main reasons for non-participation (such as failure to ask about participation, communication barriers, or complications during labour). Full details of the study design were published previously [9].

Medical records of the hospital were used to retrieve information on newborns’ sex, gestational age, date of delivery, maternal age, maternal pre-pregnancy BMI, maternal weight gain during the pregnancy, and gestational hypertension. Gestational age was determined based on the mother’s last menstrual period in combination with ultrasound data. Mothers completed questionnaires, which provided us with detailed information about socio-demographic and lifestyle factors such as parity, newborns’ ethnicity, maternal education, household smoking habits, vitamin use, and consumption of alcoholic beverages, fish, and fruit and vegetables.

In the present study, 500 bio-banked placentas were randomly selected from 799 eligible mother-neonate pairs who were recruited between March 1st, 2013, and April 1st, 2017. 462 participants remained for statistical analysis after the exclusion of failed iodine extractions (n = 2), children born before 37 weeks of pregnancy (n = 3), mothers with previous thyroid complications (i.e. hypo- [n = 12] or hyperthyroidism [n = 4]), pre-eclamptic pregnancies (n = 8), or missing data on fruit and vegetable consumption (n = 9).

### Placental sample collection and iodine analysis

The method we used for sampling and iodine determination was published previously [8]. In short, placentas were collected within 10 min after delivery and deep-frozen at -20 °C. After thawing, placental biopsies were taken at three standardized locations, situated at 2 cm from the umbilical cord. Membranes were trimmed away and excess blood was removed by rubbing it against a Grade 54 filter paper (GE Healthcare, Chicago, United States). The tissue sample of each location was stored in metal-free containers (Sterile propylene tubes; VWR, Pennsylvania, United States) and was kept frozen at − 20 °C until extraction. Extraction was done by heating 500 mg placental tissue (consisting of an equal weight of each of the 3 sampling locations) in 0.5% tetramethylammonium hydroxide (TMAH) for 3 h at 90 °C in a DigiPREP block digestion system (SCP SCIENCE, Quebec, Canada). Iodine was measured by ICP-MS (Elan DRC II; Perkin Elmer, Massachusetts, United States) on mass ^127^I, with ^125^Te as an internal standard.

### Blood collection and thyroid hormone measurements

Plastic BD Vacutainer® Lithium Heparin Tubes (BD, Franklin Lakes, New Jersey, USA) were used to collect 8 mL of cord blood immediately after delivery and 8 mL of maternal blood at one day after delivery via venepuncture. Plasma from these samples was obtained after centrifugation at 3,200 rpm for 15 min and was frozen at − 80 °C. At the clinical laboratory of the East-Limburg Hospital, the plasma levels of the thyroid hormones FT_4_ (pmol/L), FT_3_ (pmol/L), and TSH (mIU/L) were measured with an electro-chemiluminescence immunoassay using the Modular E170 automatic analyser (Roche, Basel, Switzerland).

### Statistical methods

Database management and statistical analysis were performed with the SAS software, version 9.4 (SAS Institute, Cary, NC, USA). Mean ± standard deviation (SD) is given for continuous variables and the proportion for categorical variables. The normality of the data distributions was tested with the Shapiro–Wilk statistic and quantile–quantile plots.

First, we assessed the distributions of continuous variables (ANOVA) and the proportions of categorical variables (*χ*^2^-statistics) across tertiles of the placental iodine concentrations.

Second, we identified determinants of placental iodine concentrations by forward stepwise linear regression procedures in which we set the p-value at 0.15 for the independent variables to enter and to stay in the model. The following 20 variables were considered: maternal age, pre-pregnancy BMI, gestational weight gain, maternal education (coded as low [no diploma or primary school], middle [high school], or high [college or university degree]), gestational hypertension (coded as yes or no), maternal smoking habits (coded as never-smoker, cessation before pregnancy, or current smoker), maternal exposure to indoor second-hand smoke (SHS; coded as non-smokers not exposed to SHS, non-smoker exposed to SHS, or current smoker), consumption of alcoholic beverages during pregnancy (coded as none or maximum one glass per day), maternal fish consumption (coded as did not consume during pregnancy, consumed fish less than once per week, or at least once per week), maternal fruit and vegetable consumption (coded as less than once per day, daily, or more than once per day), multi-vitamin use (coded as yes or no), anti-inflammatory medication use (coded as yes or no), blood pressure medication use (coded as yes or no), antibiotics use (coded as yes or no), gestational age, neonate’s sex, parity (coded as one, two, or at least three children), ethnicity [classified on the basis of the native country of the neonates’ grandparents as either European (at least two grandparents were European) or non-European (at least three grandparents were of non-European origin)], date of delivery as a proxy for the time trend, and season of delivery [coded as winter (December 21st to March 20th), spring (March 21st to June 20th), summer (21st to September 20nd), or autumn (September 21st to December 20th)]. Effect estimates are presented for the final model with the determinants selected by the stepwise selection procedure.

Third, to evaluate the biological significance of our placental iodine findings, we measured the plasma FT_4_, FT_3_, and TSH hormone levels in cord blood and maternal blood. After exclusion of outliers (n = 6), and missing blood samples for either child or mother (n = 78), a total of 378 participants remained for the thyroid hormone analyses. We assessed the associations between these hormones and placental iodine with a linear regression model, adjusted for common variables such as the neonate’s sex, birth weight, gestational age, parity, maternal pre-pregnancy BMI, maternal age at delivery, maternal education, and maternal smoking status (Model A) as tested previously [11], or for the determinants selected by the stepwise selection procedure (Model B).

In sensitivity analyses, we re-ran the stepwise linear regression model with the same twenty variables, except for pre-pregnancy BMI and gestational weight gain, which were in the sensitivity analysis recoded into a new variable based on the United States Institute of Medicine recommendations on the maternal gestational weight gain [12]. Recoding comprised: ‘low weight gain’ (n = 103), ‘normal weight gain’ (n = 146), and ‘excess weight gain’ (n = 213). For underweight women, the ‘normal weight gain’ is between 12.5 and 18 kg; for normal, overweight, and obese women, the ranges are 11.5–16 kg, 7–11.5 kg, and 5–9 kg, respectively. Women who gained weight below these ranges were categorized as ‘low weight gain’, while those who gained more weight were categorized as ‘excess weight gain’ [12]. In further sensitivity analyses, we excluded women who consumed any alcoholic beverages during their pregnancy (n = 62) or those who smoked during the pregnancy (n = 42).

## Results

### Population characteristics

Table 1 shows the sociodemographic, lifestyle and clinical characteristics of the participants (n = 462); the characteristics for 378 participants, whose thyroid hormones were measured, did not significantly differ from the total study population (Additional file 1: Table S1). Mothers were, on average (SD), 29.5 years old (4.4). The mean maternal pre-pregnancy BMI was 24.5 kg/m^2^ (4.7), and mothers gained on average 13.8 kg (5.7) during their pregnancy. Most of the mothers had a BMI between 18.5 and 25 kg/m^2^ before their pregnancy (61.3%), while a third of the population was overweight (22.7%) or obese (13.0%), and 14 mothers (3.0%) were underweight. The majority of the mothers obtained a higher education (54.1%) and 64.5% of all participants never smoked. During their pregnancy, twenty-six participants (5.6%) were exposed to indoor SHS, and over half of the mothers took multi-vitamins (56.1%). A total of 62 women consumed alcoholic beverages during pregnancy (13.4%). Most of them consumed less than one glass per week (n = 54), and eight consumed at least one glass per week during gestation (with a maximum of two consumptions per week). Median consumption during the entire pregnancy was seven glasses of alcoholic beverages, with an interquartile range from 2 to 18 glasses.

**Table 1:** Sociodemographic, lifestyle, and clincial characteristics of the 462 mother-neonate pairs and by tertiles of placental iodine concentration

Characteristics	Total (n = 462)	Tertile 1 (n = 154)	Tertile 2 (n = 154)	Tertile 3 (n = 154)	*p* value for trend
Mother					
Age, years	29.5 (4.4)	29.3 (3.9)	29.5 (4.8)	29.6 (4.5)	0.75
Pre-pregnancy BMI, kg/m^2^	24.5 (4.7)	24.8 (5.0)	24.6 (4.6)	24.2 (4.7)	0.54
Gestational weight gain, kg	13.8 (5.7)	14.5 (5.7)	13.9 (5.6)	13.0 (5.9)	0.07
Hypertension					0.35
Yes	27 (5.8%)	6 (3.9%)	12 (7.8%)	9 (5.8%)	
Smoking status					0.08
Never-smoker	298 (64.5%)	87 (56.5%)	107 (69.5%)	104 (67.5%)	
Cessation before pregnancy	122 (26.4%)	53 (34.4%)	32 (20.8%)	37 (24.0%)	
Current smoker	42 (9.1%)	14 (9.1%)	15 (9.7%)	13 (8.5%)	
Exposure to indoor second-hand smoke					0.32
Yes	26 (5.6%)	12 (7.8%)	6 (3.9%)	8 (5.2%)	
Alcohol consumption^a^					0.50
None	400 (86.6%)	130 (84.4%)	133 (86.4%)	137 (89.0%)	
≤2 glasses per week	62 (13.4%)	24 (15.6%)	21 (13.6%)	17 (11.0%)	
Education^b^					0.58
Low	59 (12.8%)	20 (13.0%)	24 (15.6%)	15 (9.7%)	
Middle	153 (33.1%)	54 (35.0%)	47 (30.5%)	52 (33.6%)	
High	250 (54.1%)	80 (52.0%)	83 (53.9%)	87 (56.8%)	
Multi-vitamin use					0.12
Yes	259 (56.1%)	77 (50.0%)	87 (56.5%)	95 (61.7%)	
Fish consumption					0.63
Never	34 (7.4%)	14 (9.1%)	8 (5.2%)	12 (7.8%)	
Less than once per week	205 (44.4%)	66 (42.9%)	74 (48.1%)	65 (42.2%)	
At least once per week	223 (48.2%)	74 (48.0%)	72 (46.8%)	77 (50.0%)	
Fruit and vegetable consumption					0.58
Less than once per day	55 (11.9%)	17 (11.0%)	16 (10.4%)	22 (14.3%)	
Once per day	146 (31.6%)	50 (32.5%)	54 (35.1%)	42 (27.3%)	
More than once per day	261 (56.5%)	87 (56.5%)	84 (54.6%)	90 (58.4%)	
Neonate					
Gestational age, weeks	39.9 (1.0)	39.8 (1.1)	39.8 (1.0)	40.1 (0.9)	0.07
Birth weight, g	3458 (426)	3472 (465)	3426 (431)	3476 (378)	0.51
Birth length, cm	50.3 (1.9)	50.4 (1.9)	50.2 (1.8)	50.4 (1.8)	0.75
Sex					0.48
Male	237 (51.3%)	77 (50.0%)	85 (55.2%)	75 (48.7)	
Ethnicity					0.78
European	403 (87.2%)	135 (87.7%)	136 (88.3%)	132 (85.7%)	
Parity					0.83
1	244 (52.8%)	79 (51.3%)	80 (52.0%)	85 (55.2%)	
2	156 (33.8%)	51 (33.1%)	53 (34.4%)	52 (33.8%)	
≥3	62 (13.4%)	24 (15.6%)	21 (13.6%)	17 (11.0%)	
Season at delivery					0.04
Winter (Dec 21 to March 20)	105 (22.7%)	24 (15.6%)	42 (27.3%)	39 (25.3%)	
Spring (March 21 to June 20)	111 (24.0%)	34 (22.0%)	44 (28.6%)	33 (21.4%)	
Summer (June 21 to Sept 20)	133 (28.8%)	48 (31.2%)	37 (24.0%)	48 (31.2%)	
Autumn (Sept 21 to Dec 20)	113 (24.5%)	48 (31.2%)	31 (20.1%)	34 (22.1%)	
Placental iodine concentration, µg/kg	26.1 (4.3)	21.6 (2.0)	25.8 (1.0)	30.8 (2.7)	-

Data are presented as mean (SD) or n (%). Tertiles are based on the placental iodine concentration: I ≤ 24.2; 24.2 < I ≤ 27.5; and I > 27.5

^a^For full details, please see the population characteristics in the results section

^b^Coded as ‘low’ (no diploma or primary school), ‘middle’ (high school) and ‘high’ (college or university degree)

^c^Classification of ethnicity is based on the native country of the neonates' grandparents as either European (at least two grandparents were European) or non-European (at least three grandparents were of non-European origin)

Of the neonates, 52.8% were primiparous and 51.3% were boys. They had a mean birth weight of 3458 g (426) and measured 50.3 cm (1.9) in length. The majority of the neonates (n = 403; 87.2%) were of European descent. The mean gestational age was 39.9 weeks (1.0). Births were spread more or less evenly over all seasons of the year.

### Determinants of placental iodine

The mean placental iodine concentration was 26.1 µg/kg (4.3), ranging from 12.4 to 40.5 µg/kg, and did not differ between newborn girls and boys (*p* = 0.48). We first examined the population characteristics across tertiles of placental iodine: only the season at delivery differed significantly between these tertiles (*p* value for trend = 0.04), while there was a borderline significance for gestational weight gain (*p* = 0.07), smoking status (*p* = 0.08), and gestational duration (*p* = 0.07).

Out of the 20 a priori chosen variables, the following 13 variables did not enter the forward stepwise regression model: maternal age, maternal education, gestational hypertension, maternal smoking habits, exposure to indoor SHS, maternal fish consumption, maternal fruit and vegetable consumption, anti-inflammatory medication use, blood pressure medication use, antibiotics use, neonate’s sex, parity, and ethnicity.

**Fig. 1:**
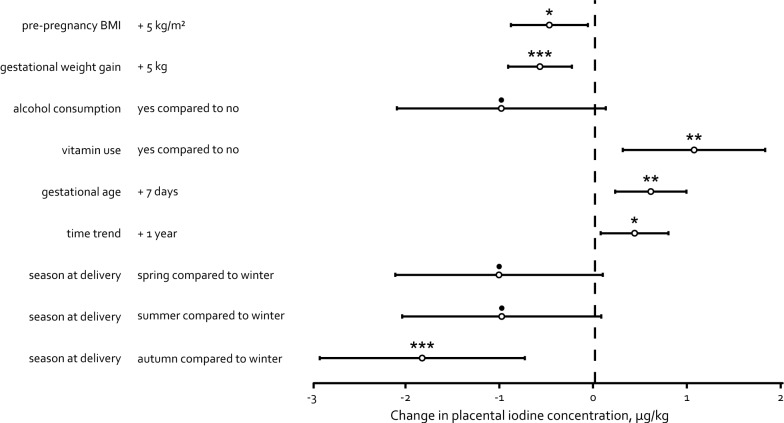
Determinants of placental iodine concentrations in multiple regression analyses (n = 462). The determinants were selected by stepwise linear regression analysis, setting the p-value for entering and to stay in the model at 0.15. In the final model, the estimate of each determinant is presented after adjustment for the other determinants. •*p* < 0.15; *p < 0.05; **p < 0.01; ***p < 0.001

Seven variables significantly determined placental iodine concentrations: i.e. pre-pregnancy BMI, gestational weight gain, alcohol consumption, vitamin use, gestational age, date at delivery, and season at delivery (Fig. 1). The effect estimate for each determinant has been calculated: an increase of 5 kg/m^2^ in maternal pre-pregnancy BMI was associated with a 0.49 µg/kg decrease in placental iodine content [95% confidence interval (CI): − 0.90–− 0.08; *p* = 0.020]. Mothers who gained on average 5 kg of weight during gestation had about a similar decrease in placental iodine content of 0.59 µg/kg (95% CI: − 0.93–− 0.25; *p* = 0.0007). Further, mothers who consumed a maximum of one glass of alcoholic beverage per day had a 1.00 µg/kg decrease in placental iodine load (95% CI: − 2.11–0.11; *p* = 0.078), while consumption of multi-vitamins during gestation was associated with a 1.06 µg/kg higher (95% CI: 0.30–1.81; *p* = 0.007) placental iodine concentration. Both gestational age and date of delivery were positively associated with placental iodine concentrations, which increased with 0.59 µg/kg (95% CI: 0.21− 0.98; *p* = 0.002) for an increase of one week in pregnancy and 0.42 µg/kg (95% CI: 0.06− 0.78; *p* = 0.022) for a year increase in date of delivery (as time trend). For seasonality, we observed the highest placental iodine concentrations for neonates born during the winter. Compared to this season, the concentrations decreased with 1.02 µg/kg (95% CI: − 2.13–0.08; *p* = 0.070), 1.00 µg/kg (95% CI: − 2.06–0.07; *p* = 0.066), and 1.84 µg/kg (95% CI: − 2.94–− 0.75; *p* = 0.001) for neonates born in spring, summer, and autumn respectively (Fig. 1).

### Association between thyroid hormones and placental iodine

**Fig. 2:**
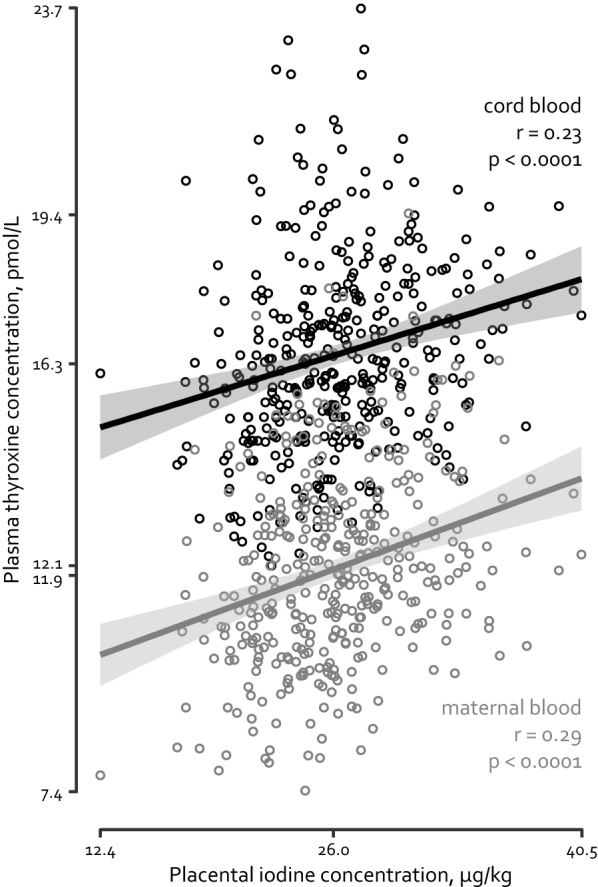
Maternal and cord plasma free thyroxine (FT_4_) concentrations are positively associated with placental iodine concentration. Pearson correlation coefficients were 0.29 (*p* < 0.0001) and 0.23 (*p* < 0.0001) for maternal and cord FT_4_ respectively. Maternal FT_4_ concentrations ranged from 7.4 to 19.4 pmol/L, with a median of 11.9 pmol/L. Cord FT4 concentrations ranged from 12.1 to 23.7 pmol/L, with a median value of 16.3 pmol/L

For 378 mother-neonate pairs the thyroid hormones FT_4_, FT_3_, and TSH were measured in cord and maternal plasma. Table 2 describes the results for the tertiles of placental iodine concentration (i.e. I < 24.2; 24.2 < I ≤ 27.5; and I > 27.5 µg/kg). Across the tertiles, only FT_4_ in mother and cord blood showed a significant increase (p < 0.0001), which is corroborated by significant Pearson correlations (Fig. 2) between placental iodine concentrations and plasma FT_4_ concentrations in maternal blood (*r* = 0.29; *p* < 0.0001) and cord blood (*r* = 0.23; *p* < 0.0001). No correlation was found between maternal and cord FT_4_ plasma levels (*r* = 0.04; *p* = 0.45). The associations calculated for Model A showed for an increase of 5 µg/kg in placental iodine, 0.64 pmol/L higher maternal FT_4_ (95% CI: 0.42–0.86; *p* < 0.0001) and a 0.64 pmol/L higher cord FT_4_ (95% CI: 0.42–0.87; *p* < 0.0001). Analysing the associations in Model B with adjustments for the determinants selected by the stepwise selection procedure slightly altered the estimates, i.e. for maternal FT_4_: 0.73 pmol/L (95% CI: 0.50–0.93; *p* < 0.0001); and for cord FT_4_ 0.61 pmol/L (95% CI: 0.37–0.85; *p* < 0.0001). Neither in Model A nor in Model B, placental iodine concentration showed significant associations with maternal FT_3_, maternal TSH, cord FT_3_, and cord TSH.

**Table 2:** Concentration of the thyroid hormones FT_4_, FT_3_, and TSH in maternal blood and cord blood of 378 mother-neonate pairs and by tertiles of placental iodine concentration

Thyroid hormone	Total (n = 378)	Tertile 1 (n = 122)	Tertile 2 (n = 125)	Tertile 3 (n = 131)	*p* value for trend
Mother					
FT_4_, pmol/L	12.1 (2.0)	11.4 (1.9)	12.0 (2.0)	12.7 (1.8)	0.0001
FT_3_, pmol/L	4.0 (1.0)	4.1 (0.9)	3.9 (1.0)	4.1 (1.2)	0.25
TSH, mIU/L	2.4 (1.3)	2.5 (1.2)	2.3 (1.4)	2.4 (1.3)	0.49
Neonate					
FT_4_, pmol/L	16.5 (2.0)	15.9 (2.2)	16.5 (1.9)	17.0 (1.9)	0.0001
FT_3_, pmol/L	2.3 (0.6)	2.3 (0.5)	2.4 (0.6)	2.4 (0.6)	0.36
TSH, mIU/L	10.7 (7.6)	10.5 (7.0)	11.2 (8.6)	10.4 (7.3)	0.67
Placental iodine concentrations, µg/kg	26.2 (4.3)	21.6 (2.0)	25.8 (1.0)	30.9 (2.8)	–

Data are presented as mean (SD). Tertiles are based on the placental iodine concentration: I ≤ 24.2; 24.2 < I ≤ 27.5; and I > 27.5

### Sensitivity analyses

In the stepwise linear regression model, where pre-pregnancy BMI and gestational weight gain were replaced with a new categorical variable, all other previously reported determinants entered the model, with minimal deviations from the earlier reported estimates (data not shown). For the newly recoded determinant, we observed a significant decrease of 1.26 µg/kg in placental iodine concentration (95% CI: − 2.13–− 0.38; *p* = 0.005) if the mothers gained an excess amount of weight during their pregnancy, compared to those with a weight gain in the normal range. No difference was found between women with a low weight gain and those with a normal weight gain.

In the stepwise multiple linear regression models in which we excluded the mothers who consumed alcohol (n = 62) or the current smokers (n = 42), all the aforementioned determinants were selected once again (Additional file 1: Table S2). Little to no variation was found for the effect estimates. However, in the subset of mothers who did not consume any alcohol, the variable sex reached the 0.15 selection threshold as well.

## Discussion

In the present cross-sectional study, out of 20 variables, we identified seven determinants of iodine concentrations in the placental tissue of mother-neonate pairs from the ENVIR*ON*AGE birth cohort. We previously reported the urinary iodine concentrations in a subset of 96 participants, which indicated a mild-to-moderate iodine deficiency in the current cohort [8]. In short, significant sociodemographic determinants of placental iodine concentrations were: pre-pregnancy BMI, gestational weight gain, and alcohol consumption were inversely associated with iodine concentrations; while multi-vitamin use, gestational age, and date at delivery showed a positive association. For the season at delivery, delivery in spring was associated with the highest placental iodine concentrations compared to delivery in spring, summer, or autumn. In a subset, we also observed significant associations of the maternal and cord FT_4_ plasma concentrations with placental iodine load.

### Pre-pregnancy BMI and gestational weight gain

In 2013, Vandevijvere and colleagues investigated the iodine status of a Belgian gestational population [10]. They observed that urinary iodine concentrations and BMI in the first trimester of pregnancy were inversely associated. Moreover, inverse associations were also found by Pop et al*.* [13] between the thyroid hormones, measured at different weeks of gestation, and pre-pregnancy BMI and gestational weight gain. Among similar lines, we showed in the present study that both pre-pregnancy BMI and gestational weight gain were inversely associated with placental iodine concentrations. Although some studies have indicated that weight gain might be a consequence of thyroid dysfunction [13, 14], a large scale prospective follow-up study of 784 adults from the south of Spain showed an inverse correlation between the urinary concentrations at baseline and weight gain over six years [15] and indicated that alterations in the thyroid hormones could be the consequence of the increase in weight rather than the cause. The exact physiological pathways linking weight gain and the iodine status need further clarification. We partially addressed this point in the sensitivity analysis by categorizing the population into three groups, based on recoding pre-pregnancy BMI and gestational weight gain. We showed that the women with excess weight gain during their pregnancy had a lower placental iodine concentration compared to the mothers with a normal weight gain.

### Alcohol consumption

Findings from the National Health and Nutrition Examination Survey (NHANES) showed that alcohol consumption (groups of alcohol consumption: ‘none’, ‘ < 1 per day’, ‘1–2 per day’, or ‘ ≥ 2 per day’) was inversely associated with the urinary iodine concentrations in adults above the age of 20, and progressively decreased with increasing amounts of alcohol consumed [16]. In contrast, Vandevijvere and colleagues reported, based on the urinary concentrations, that pregnant women who consumed alcohol had a lower risk of iodine deficiency [10]. It should be noted that both these contradicting observations concern the urinary iodine concentrations, which is a short-term biomarker of iodine intake. In the present study, we observed that women who consumed alcohol during pregnancy had a lower placental iodine concentration than women who refrained from drinking alcoholic beverages, which corroborates the results of the NHANES study. Our findings suggest that long-term storage of placental iodine might become compromised by the consumption of alcoholic beverages during pregnancy, even at small amounts of alcohol intake (i.e. maximally one glass per week). The exact mechanism of how alcohol can influence the urinary and placental iodine concentrations remains unclear.

### Multi-vitamin use

In 2007, the Norwegian mother and child cohort (MoBa) observed that iodine in urine was higher in users of iodine-containing multi-vitamin than in those who consumed non-iodine-containing supplements [17]. Furthermore, Zimmermann and Delange [1] reviewed seven studies (published between 1991 and 2002) and observed that all of them showed increased urinary concentrations after iodine supplementation during pregnancy. Our findings complement these studies as we showed for the first time that supplementation with multi-vitamins during pregnancy positively affects the long-term iodine storage in placental tissue.

### Gestational age

It has been previously reported that urinary iodine decreases during the last stages of pregnancy, which would represent depletion of the iodine stores due to fetoplacental use, an increased GFR-related loss, and inadequate dietary compensation [18]. This inverse association was also observed by others [19, 20], whereas some authors demonstrated a positive association between urinary iodine concentrations and gestational age [10, 21]. Unlike all the aforementioned studies, which focused on the urinary concentrations of iodine, our investigations involved placental iodine concentrations measured at the very end of the pregnancy (i.e. from 37 until 41 weeks of gestation). We found a positive association between gestational age and placental iodine concentrations. This might imply changes in foetal iodine supply from the placenta, perhaps because the foetal demands for iodine are lower at the very end of gestation, however, the exact mechanism is still to be unravelled.

### Time trend

In April 2009, an agreement was signed between the Belgian Ministry of Health and the bakery sector, to stimulate the fortification of bread with iodized salt [22]. Salt iodization is known to be a successful type of food fortification. In 2014, 36.2% of the Belgian population indicated to use iodized salt when cooking [23]. Several studies have shown a positive effect of salt iodization on the iodine status of populations [1, 24, 25]. The observed increase in placental iodine of 0.42 µg/kg per year, between 2013 and 2017, may suggest that a continued positive effect of the fortification campaign is present, or that household use of iodized salt is increasing.

### Seasonality

Several studies in other European countries, showed that urinary iodine concentrations are lower in the summer months and highest during winter [25, 26]. The Swiss longitudinal pilot study [26] found seasonal differences in pre-school children (average urinary iodine concentration of 144 µg/L), but not in adults (average urinary concentration of 96 µg/L). Furthermore, the Danish cohort from Rasmussen et al*.* [25] observed similar seasonal differences in an adult population (median urinary iodine concentration was 93 µg/24 h for Copenhagen and 62 µg/24 h for Aalborg). A more recent investigation by Vandevijvere et al. [10] in Belgian pregnant women (median urinary iodine concentration of 125.1 µg/L) showed that the risk at iodine deficiency was highest in autumn, compared to winter. In line with these studies, we observed in our study a seasonal effect for placental iodine concentrations, as children born in the winter had the highest placental iodine concentrations compared to any other season. It is well documented that seasonality plays an important role in the iodine levels of milk and dairy products, which are higher during the winter months because the cattle are more often housed indoors and fed with iodine-enriched feed or feed supplements [22]. Since milk is considered to be an important dietary source of iodine in Belgium [27], the seasonal variation in placental iodine concentrations observed in our study might be attributed to the consumption of dairy with higher concentrations of iodine.

### Thyroid hormones and placental iodine

We assessed the biological significance of placental iodine concentration in the context of the measured thyroid hormones. Although no associations between plasma FT_3_ or TSH and placental iodine were observed, significant correlations were revealed between placental iodine concentration and plasma FT_4_ in both maternal and cord blood. FT_4_ is the most abundant form of thyroid hormone in blood and coincidentally it has the longest half-life of all thyroid hormones, serving as a precursor of the active T_3_ form. An inadequate supply of FT_4_ to the neonate’s developing neural system seems to underlie the adverse cognitive and/or behavioural development such as delayed mental and motor function [28], attention deficit and hyperactivity disorder [3], lower IQ [3, 29], and subjects with extremely low FT_4_ concentrations (i.e. < 3^rd^ percentile) are at a higher risk of autism spectrum disorders [30]. These observations, therefore, confirm the biological significance of placental iodine levels because functional effects of lower FT_4_ have previously been reported.

### Limitations and strengths

We acknowledge some limitations in the current study. First, detailed nutritional information is absent for the entire study population. We used two simplified variables (i.e. fish, and fruit and vegetable consumption) as a proxy for dietary iodine intake. A detailed food frequency questionnaire was included at a later stage of the recruitment and was subsequently available for only 97 participants. Nevertheless, re-analysing the models in this subset (with the addition of the following variables: consumption of vegetables, meat, cheese, yoghurt, milk, fish, seaweed, and cereals) showed similar results as the total population (data not shown). Second, we cannot account for possible bias of self-reported variables like alcohol consumption and smoking habits. Therefore, in a sensitivity analysis, we excluded alcohol-consuming participants or current smokers and could conclude that our findings remained unaltered. Measurements of plasma cotinine levels in a limited group (n = 40) indicated that our classification of ‘smoker’ – ‘non-smoker’ did not suffer from bias (data not shown). Finally, the cross-sectional observational study design did not allow us to draw causal conclusions.

A major strength of the study is that our population is representative of the segment of the Belgian population in the pregnancy period or reproductive phase of life, suggesting that our findings are generalizable (Additional file 1: Table S3) [31]. Secondly, repeatability, reproducibility, and variation of placental iodine concentrations between the sample regions were adequately checked previously [8]. Furthermore, our results remained robust in several sensitivity analyses.

## Conclusion

The current study sheds light on possible determinants of long-term iodine storage as evaluated for placental tissue. Maternal factors linked with lower placental iodine concentrations were: a higher pre-pregnancy BMI, higher gestational weight gain, and alcohol consumption. Multi-vitamin supplementation during pregnancy was associated with higher levels of iodine in the placenta. Lastly, the highest concentrations of placental iodine were found for children born during the winter period. The current study, therefore, identifies several factors that can alter the iodine stores during the gestational period of life. Of biological importance is that the maternal and cord plasma FT_4_ concentrations positively associated with the placental iodine concentrations. Future studies should elucidate the effects of altered placental iodine storages on neonatal health and subsequently on health later in life.

## Supplementary information

**Additional file 1:** **Table S1.** Maternal and neonate characteristics in the current study (n=462), and those with thyroid hormone data (n=378). **Table S2. **Determinants of placental iodine concentrations in multiple regression analysis for the current study (n=462), the non-smokers (n=420), or those who did not consume any alcohol (n=400). **Table S3. **Maternal and neonate characteristics of the current study group (n=462) compared with a reference population of births in Flanders, Belgium (born 2002 until 2011; n=606,877).

## Data Availability

The datasets used and analysed during the current study are available from the corresponding author on reasonable request.

## Abbreviations

BMI: Body mass index
ENVIR*ON*AGE: ENVIRonmental influences *ON* early AGEing
FT_3_: Free tri-iodothyronine
FT_4_: Free thyroxine
NHANES: National Health and Nutrition Examination Survey
SD: Standard deviation
SHS: Second-hand smoke
TMAH: Tetramethylammonium hydroxide
TSH: Thyroid-stimulating hormone
