# Supplementary material for: Determinants of placental iodine concentrations in a mild-to-moderate iodine-deficient population: an ENVIRONAGE cohort study
Source: J Transl Med. 2020 Nov 10;18:426. doi: 10.1186/s12967-020-02601-8 (PMC7654607; doi:10.1186/s12967-020-02601-8)
Supplement: Supplementary file 1 — Additional file 1: Table S1. Maternal and neonate characteristics in the current study (n=462), and those with thyroid hormone data (n=378). Table S2. Determinants of placental iodine concentrations in multiple regression analysis for the current study (n=462), the non-smokers (n=420), or those who did not consume any alcohol (n=400). Table S3. Maternal and neonate characteristics of the current study group (n=462) compared with a reference population of births in Flanders, Belgium (born 2002 until 2011; n=606,877). [file 12967_2020_2601_MOESM1_ESM.docx]

**Supplemental Table 1: Maternal and neonate characteristics** **in the current study (n=462), and those with thyroid hormone data (n=378).**

| **Characteristics** | **Total group (n=462)** | **Thyroid hormones available  (n=378)** | **p-value** |
| --- | --- | --- | --- |
| **Maternal** |  |  |  |
| Age, years | 29.5 (4.4) | 29.5 (4.4) | 0.89 |
| Pre-pregnancy BMI, kg/m^2^ | 24.5 (4.7) | 24.1 (4.4) | 0.20 |
| Net weight gain, kg | 13.8 (5.7) | 13.9 (5.5) | 0.93 |
| Hypertension |  |  | 0.73 |
| *Yes* | 27 (5.8%) | 20 (5.3%) |  |
| Smoking status |  |  | 0.96 |
| *Never-smoker* | 298 (64.5%) | 244 (64.6%) |  |
| *Cessation before pregnancy* | 122 (26.4%) | 98 (25.9%) |  |
| *Current smoker* | 42 (9.1%) | 36 (9.5%) |  |
| Exposure to indoor second-hand smoke |  |  | 0.58 |
| *Yes* | 26 (5.6%) | 18 (4.8%) |  |
| Alcohol consumption |  |  | 0.94 |
| *None* | 400 (86.6%) | 328 (86.8%) |  |
| *≤ 1 glass per day* | 62 (13.4%) | 50 (13.2%) |  |
| Education^a^ |  |  | 0.90 |
| *Low* | 59 (12.8%) | 51 (13.5%) |  |
| *Middle* | 153 (33.1%) | 120 (31.8%) |  |
| *High* | 250 (54.1%) | 207 (54.8%) |  |
| Vitamin use |  |  | 0.56 |
| *Yes* | 259 (56.1%) | 219 (57.9%) |  |
| Fish consumption |  |  | 0.83 |
| *Never* | 34 (7.4%) | 32 (8.5%) |  |
| *Less than once per week* | 205 (44.4%) | 167 (44.2%) |  |
| *At least once per week* | 223 (48.2%) | 179 (47.4%) |  |
| Fruit and vegetable consumption |  |  | 0.90 |
| *Less than once per day* | 55 (11.9%) | 47 (12.4%) |  |
| *Once per day* | 146 (31.6%) | 114 (30.2%) |  |
| *More than once per day* | 261 (56.5%) | 217 (57.4%) |  |
| **Neonate** |  |  |  |
| Gestational age, weeks | 39.9 (1.0) | 39.9 (1.0) | 0.73 |
| Birth weight, g | 3458 (426) | 3450 (422) | 0.77 |
| Birth length, cm | 50.3 (1.9) | 50.3 (1.9) | 0.89 |
| Sex |  |  | 0.98 |
| *Male* | 237 (51.3%) | 194 (51.3%) |  |
| Ethnicity^b^ |  |  | 0.92 |
| *European* | 403 (87.2%) | 329 (87.0%) |  |
| Parity |  |  | 0.97 |
| *1* | 244 (52.8%) | 202 (53.4%) |  |
| *2* | 156 (33.8%) | 127 (33.6%) |  |
| *≥ 3* | 62 (13.4%) | 49 (13.0%) |  |
| Season at delivery |  |  | 0.99 |
| *Winter (Dec 21 to March 20)* | 105 (22.7%) | 87 (23.0%) |  |
| *Spring (March 21 to June 20)* | 111 (24.0%) | 89 (23.5%) |  |
| *Summer (June 21 to Sept 22)* | 133 (28.8%) | 109 (28.8%) |  |
| *Autumn (Sept 23 to Dec 20)* | 113 (24.5%) | 93 (24.6%) |  |
| Placental iodine concentration, µg/kg | 26.1 (4.3) | 26.2 (4.3) | 0.66 |

*Data are presented as mean (SD) for continuous variables, and as n (%) for discrete variables.*

*The distributions of continuous variables (ANOVA) and the proportions of categorical variables (χ²-statistics) were assessed between the total population and the subset for which thyroid hormone data was available.*

*a Coded as ‘low’ (no diploma or primary school), ‘middle’ (high school) or ‘high’ (college or university degree).
b Classification of ethnicity is based on the native country of the neonates' grandparents as either European (at least two grandparents were European) or non-European (at least three grandparents were of non-European origin).*

**Supplemental Table 2: Determinants of placental iodine concentrations in multiple regression analysis for the current study (n=462), the non-smokers (n=420), or those who did not consume any alcohol (n=400).**

|  | **Total group (n=462)** | | |  | **Non-smokers (n=420)** | | |  | **No alcohol use (n=400)** | | |
| --- | --- | --- | --- | --- | --- | --- | --- | --- | --- | --- | --- |
| **Independent variables** | **Estimate** | **95% CI** | **p-value** |  | **Estimate** | **95% CI** | **p-value** |  | **Estimate** | **95% CI** | **p-value** |
| Pre-pregnancy BMI (+5 kg/m^2^) | -0.49 | -0.90 to -0.08 | 0.020 |  | -0.46 | -0.88 to -0.03 | 0.034 |  | -0.59 | -1.02 to -0.15 | 0.008 |
| Gestational weight gain (+5 kg) | -0.59 | -0.93 to -0.25 | 0.0007 |  | -0.53 | -0.89 to -0.17 | 0.004 |  | -0.65 | -1.01 to -0.29 | 0.0005 |
| Alcohol consumption^a^ | -1.00 | -2.11 to 0.11 | 0.078 |  | -1.08 | -2.23 to 0.08 | 0.068 |  | . | . | . |
| Vitamin use^b^ | 1.06 | 0.30 to 1.81 | 0.007 |  | 1.16 | 0.36 to 1.96 | 0.005 |  | 1.06 | 0.24 to 1.87 | 0.011 |
| Gestational age (+1 week) | 0.59 | 0.21 to 0.98 | 0.002 |  | 0.45 | 0.04 to 0.86 | 0.030 |  | 0.56 | 0.15 to 0.98 | 0.008 |
| Date at delivery (+ 365 days) | 0.42 | 0.06 to 0.78 | 0.022 |  | 0.39 | 0.02 to 0.77 | 0.413 |  | 0.46 | 0.07 to 0.86 | 0.021 |
| Season at delivery^c^ |  |  |  |  |  |  |  |  |  |  |  |
| *Spring (Mar 21^st^ – Jun 20^th^)* | -1.02 | -2.13 to 0.08 | 0.070 |  | -1.05 | -2.22 to 0.12 | 0.079 |  | -0.96 | -2.15 to 0.23 | 0.114 |
| *Summer (Jun 21^st^ – Sep 20^th^)* | -1.00 | -2.06 to 0.07 | 0.066 |  | -0.95 | -2.06 to 0.17 | 0.097 |  | -1.07 | -2.23 to 0.08 | 0.068 |
| *Autumn (Sep 21^st^ – Dec 20^th^)* | -1.84 | -2.94 to -0.75 | 0.001 |  | -1.88 | -3.05 to -0.72 | 0.002 |  | -1.92 | -3.09 to -0.74 | 0.001 |
| Sex of neonate^$^ | . | . | . |  | . | . | . |  | -0.69 | -1.50 to 0.13 | 0.099 |

*Determinants of placental iodine concentrations were selected by stepwise linear regression analysis, setting the p-value for entering and to stay in the model at 0.15.*

*Estimates for the presented determinants are adjusted for the other variables in the final model.*

*a “Yes” compared to “No” in a given question.*

*b Seasons compared to Winter (Dec 21^st^ – Mar 20^th^).*

*c “Male” compared to “Female”.*

**Supplemental Table 3: Maternal and neonate characteristics** **of the current study group (n=462) compared with a reference population of births in Flanders, Belgium (born 2002 until 2011; n=606,877).**

| **Characteristics** | **Current study (n=464)** | **Births in Flanders (n=606,877)^a^** |
| --- | --- | --- |
| **Maternal** |  |  |
| Age, years | 29.5 (24.0 – 35.0) | 29.5 (23.5 – 35.8) |
| < 25 | 48 (10.4%) | 98,419 (16.2%) |
| 25 – 34 | 340 (73.4%) | 428,781 (70.7%) |
| 35+ | 74 (16.2%) | 79,677 (13.1%) |
| Maternal education^b^ |  |  |
| *Low* | 59 (12.8%) | 58,743 (13.1%) |
| *Middle* | 153 (33.1%) | 183,410 (40.8%) |
| *High* | 250 (54.1%) | 207,563 (46.2%) |
| **Newborn** |  |  |
| Birth weight, g | 3,458 (2,870 – 4,015) | 3,360 (2,740 – 3,965) |
| Sex |  |  |
| *Male* | 237 (51.3%) | 311,620 (51.4%) |
| Ethnicity^c^ |  |  |
| *European* | 403 (87.2%) | 384,522 (87.7%) |
| Parity |  |  |
| *1* | 244 (52.8%) | 284,770 (46.9%) |
| *2* | 156 (33.8%) | 210,731 (34.7%) |
| *≥ 3* | 62 (13.4%) | 111,376 (18.4%) |
| Month of birth |  |  |
| *January* | 42 (9.1%) | 50,929 (8.4%) |
| *February* | 27 (5.8%) | 46,524 (7.7%) |
| *March* | 32 (6.9%) | 51,541 (8.5%) |
| *April* | 25 (5.4%) | 50,035 (8.2%) |
| *May* | 42 (9.1%) | 50,771 (8.4%) |
| *June* | 50 (11.0%) | 50,975 (8.4%) |
| *July* | 39 (8.4%) | 53,436 (8.8%) |
| *August* | 48 (10.6%) | 53,366 (8.8%) |
| *September* | 40 (8.6%) | 51,702 (8.5%) |
| *October* | 37 (8.0%) | 51,397 (8.5%) |
| *November* | 42 (9.1%) | 47,033 (7.8%) |
| *December* | 38 (8.2%) | 49,168 (8.1%) |

*Data are presented as median (10^th^ percentile – 90^th^ percentile) for continuous variables, and as n (%) for discrete variables.*

*^a^Cox, B., E. Martens, B. Nemery, J. Vangronsveld and T. S. Nawrot (2013). "Impact of a stepwise introduction of smoke-free legislation on the rate of preterm births: analysis of routinely collected birth data." BMJ 346: f441.*

*^b^ Coded as ‘low’ (no diploma or primary school), ‘middle’ (high school) or ‘high’ (college or university degree).*

*^c^ Classification is based on the native country of the neonates' grandparents as either European (at least two grandparents were European) or non-European (at least three grandparents were of non-European origin.*
